# Problems, principles and progress in computational annotation of NMR metabolomics data

**DOI:** 10.1007/s11306-022-01962-z

**Published:** 2022-12-05

**Authors:** Michael T. Judge, Timothy M. D. Ebbels

**Affiliations:** Section of Bioinformatics, Division of Systems Medicine, Department of Metabolism, Digestion and Reproduction, Imperial College, 131 Sir Alexander Fleming Building, South Kensington Campus, London, UK

**Keywords:** NMR metabolomics, Metabolite identification, Spectral comparison, Feature, Reference database matching, Computational annotation

## Abstract

**Background:**

Compound identification remains a critical bottleneck in the process of exploiting Nuclear Magnetic Resonance (NMR) metabolomics data, especially for ^1^H 1-dimensional (^1^H 1D) data. As databases of reference compound spectra have grown, workflows have evolved to rely heavily on their search functions to facilitate this process by generating lists of potential metabolites found in complex mixture data, facilitating annotation and identification. However, approaches for validating and communicating annotations are most often guided by expert knowledge, and therefore are highly variable despite repeated efforts to align practices and define community standards.

**Aim of review:**

This review is aimed at broadening the application of automated annotation tools by discussing the key ideas of spectral matching and beginning to describe a set of terms to classify this information, thus advancing standards for communicating annotation confidence. Additionally, we hope that this review will facilitate the growing collaboration between chemical data scientists, software developers and the NMR metabolomics community aiding development of long-term software solutions.

**Key scientific concepts of review:**

We begin with a brief discussion of the typical untargeted NMR identification workflow. We differentiate between annotation (hypothesis generation, filtering), and identification (hypothesis testing, verification), and note the utility of different NMR data features for annotation. We then touch on three parts of annotation: (1) generation of queries, (2) matching queries to reference data, and (3) scoring and confidence estimation of potential matches for verification. In doing so, we highlight existing approaches to automated and semi-automated annotation from the perspective of the structural information they utilize, as well as how this information can be represented computationally.

## Introduction

### NMR in metabolomics and compound identification

Metabolomics has become a key component of modern biological and biomedical studies, providing rich information on an organism’s biological status in health and disease. However, to exploit its full potential, the field must address the fundamental problem of metabolite identification (Edison et al., 2021; Garcia-Perez et al., 2020; Monge et al., 2019). Metabolomic assays capturing the widest range of metabolites (untargeted approaches) yield many unidentified features (spectral elements defined across samples), each of which reports on one or more small molecules. Without identification, it is extremely difficult to investigate and understand the biological mechanisms at work, either by expert interpretation or by bioinformatic approaches such as pathway analysis.

**Fig. 1:**
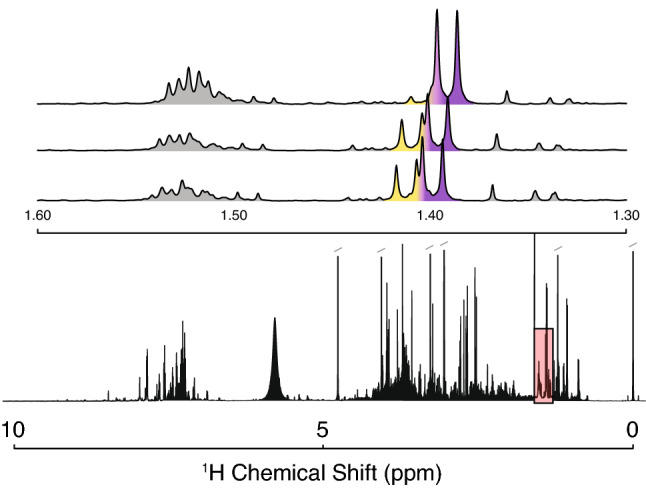
Overlap, peak shifting, and spectral crowding are major issues for NMR annotation. A full ^1^H-NMR spectrum of human urine (Salek et al., 2007; lower panel) shows the great diversity in signal intensity and shape, as well as crowded regions of the spectrum (e.g. ~ 3–4 ppm, 7–8 ppm) typical in biofluid data. Expansion (top panel) of the red box shows shifting of two doublet peaks (yellow and purple), causing them to overlap by different amounts in different samples. As a result, the observed peak shapes differ across samples, greatly complicating annotation and quantification. A collection of signals at 1.50 ppm exhibits even more complex peak shapes and overlap. Data are from study MTBLS1 at the MetaboLights repository (www.ebi.ac.uk/metabolights/MTBLS1; Haug et al., 2020)

NMR is widely accepted as one of the most powerful structural assignment tools available to the analytical chemist, yielding structural information on a wide range of levels for a huge range of molecules. Here, we focus on small molecules, but lipid and protein signals are also commonly detected. Despite these strengths, NMR is faced with several challenges, illustrated in Fig. 1, including overlap (two peaks occupying the same spectral region), peak shifting (due to pH or metal ions; Tredwell et al., 2016), relatively low sensitivity compared to mass spectrometry, spectral crowding, and complex peak shapes (discussed in more detail below). Further complications can arise from differences in resolution, field strength, and line shape across samples or studies. Full utilization of ^1^H 1D data requires expert consideration of these variables.

**Fig. 2:**
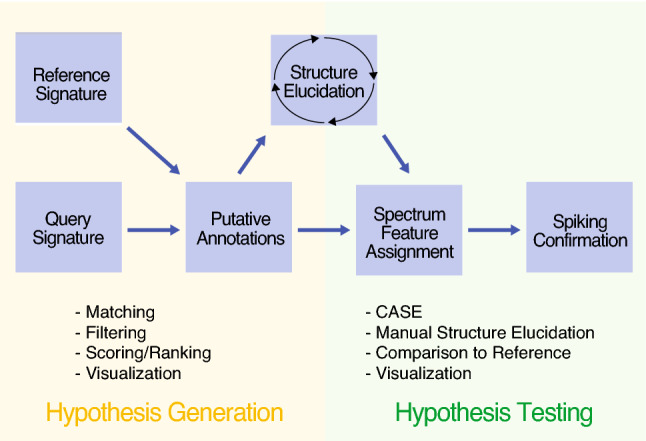
A computationally-guided compound identification workflow. Compound identification can be split into hypothesis generation and testing. First, a query (experimental) signature is generated, then it is compared computationally to reference entries for similarity. A list of putative annotations is then filtered and ranked. If this fails, a structure elucidation pipeline must be employed, typically with great cost. Once high-quality annotations are obtained, atom-level mapping is done either manually or by Computer Assisted Structure Elucidation (CASE) approaches to account for all observed data features, and spiking of a reference standard into the biological matrix is performed for full confirmation

As such, identification of compounds in metabolomic NMR spectra is a nontrivial process which is hard to automate. Unambiguous structural identification typically requires highly time-consuming examination by a field expert who can leverage the rich and nuanced theoretical concepts involved. In theory, any spectrum should be computable from first principles, and there is an excellent literature covering the identification of small molecules by NMR techniques for metabolomics and natural products research (Beniddir et al., 2021; Bingol et al., 2016; Dona et al., 2016; Garcia-Perez et al., 2020; Ellinger et al., 2013; Pauli et al., 2014; van der Hooft, Rankin 2016). We will not discuss these in detail here; instead we give an overview of the general annotation/identification process as shown in Fig. 2. Here we conceptualize a two-step process of generation of hypothesized annotations by comparing experimental and reference signatures, and hypothesis testing by manual, computational and experimental means. A common theme among annotation pipelines is that additional experimentation and final confirmation by comparison to an authentic standard are usually required for a positive and unique identification.

### Annotation as a subprocess of identification

On the other hand, annotation, the assignment of putative candidates to an observed feature using physicochemical properties or spectral similarity and metabolite databases, has become an important step towards final identification (Eghbalnia et al., 2017; Everett, 2015; Monge et al., 2019; Sumner et al., 2007; Ulrich et al., 2019). While annotation does not provide unique and certain identifications, annotations are a first step and their confidence should be expressed on an appropriate scale (Joesten, Kennedy 2019; Sumner et al., 2007). Moreover, the information obtained by annotation methods is often suitable for large-scale biological hypothesis generation. Since this process is less stringent and scale is important, it is sensible to automate annotation when an acceptable balance between confidence and scalability exists.

### Challenges in the automation of annotation

Annotation is also not easy to automate, however. Many difficulties can be traced to the complex nature of the mixtures analysed in metabolomics, as well as the relative lack of sensitivity and resolution of NMR compared to other analytical techniques. Once again, we point to excellent discussions of these issues in previous reviews (Beniddir et al., 2021). Instead, we aim to differentiate between the various computational approaches to automating annotation. Numerous additional difficulties and ambiguities in automation emerge from the implicit application of the knowledge and information the seasoned spectroscopist brings to a spectrum. We therefore find that it is helpful to delineate these approaches by the type(s) of spectral features used, the underlying structural information they utilize, and their computational characteristics. Note that in discussing these types of information we do not intend to replace long-standing terms used by the NMR community; rather, our intent is to suggest nomenclature which refers to how these elements are computationally derived and used in practical annotation. Furthermore, we will not attempt a comprehensive and rigorous assessment of available annotation tools, and point the reader to existing reviews documenting and carefully discussing existing tools (Beniddir et al., 2021; Misra, 2021).

## Data, feature complexity, and structural information

**Fig. 3:**
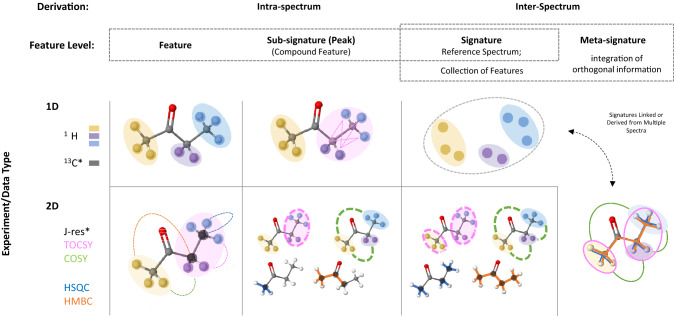
Levels of information on which matches can be based in 1D and 2D NMR annotation. Columns correspond to levels of feature complexity, and rows correspond to NMR experiment types. Different types of structural information are conveyed at the intersections, and different data characteristics apply to each. Yellow, purple, and blue colored ellipses shade chemically equivalent protons, colored respectively. ^13^C is shown in dark gray as it is a commonly probed nucleus. Pink ellipses and lines show spin system relationships, and are shown when a spin system is relevant. Data characteristics on which spectral comparisons can be made are detailed in the text. Information given by common 2D experiments are shown in respective colors. The relationships shown here are illustrative examples of the types of connections usable for pattern matching; we do not show comprehensive assignment of the example molecule

**Fig. 4:**
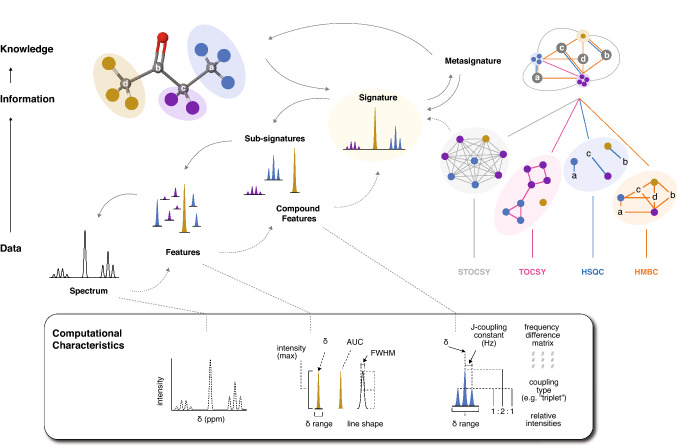
Extraction and computational representation of features. The complexity of features is related to the amount of information used (solid lines) or assumed (dashed lines) to produce them. In annotation, matching is based on computationally definable characteristics which depend on feature complexity. Examples of characteristics are illustrated in the bottom row. Colored circles indicate chemically distinct protons or signals derived from them (in the 2D case). Statistical Total Correlation Spectroscopy (STOCSY) or similar correlation-based relationships can be incorporated into a metasignature. A reference spectrum (arrow from molecule) signature can confidently be incorporated into any level of information. Letters in networks derived from 2D data indicate chemically distinct ^13^C atoms. Total Correlation Spectroscopy (TOCSY); Heteronuclear Single-Quantum Correlation (HSQC); Homonuclear Multiple Bond Correlation (HMBC); δ (chemical shift in parts per million); AUC (Area Under the Curve); FWHM (Full Width at Half Maximum)

Spectral elements (objects in a spectrum) are defined from multiple perspectives. First, the structural relationships revealed by different NMR experiments result in spectral elements of varying complexity (Table 1; Fig. 3). Next, these features are extracted and defined using characteristics which depend on that complexity; these in turn inform how features are represented computationally (box in Fig. 4). In most cases, the full range of computational characteristics are not used, and even complex features are reduced to one or two characteristics.

**Table 1:** Examples of features at different levels for butanone

Spectral element	Chemical shift ∂ (ppm)
Feature (Resonance)	2.42
Compound Feature	2.42, 2.43, 2.45, 2.47
Subsignature	2.14, 2.42, 2.43, 2.45, 2.47
Signature	1.07, 1.06, 1.04, 2.14, 2.42, 2.43, 2.45, 2.47
Metasignature	(∂^1^H) ^1^H 1D (1.07, 1.06, 1.04) coupled to (2.42, 2.43, 2.45, 2.47), 2.14 not coupled (∂^1^H, ∂^1^H) COSY (1.0, 2.5) provides coupling information

Each feature can be described using a list of constituent resonance frequencies (approximate chemical shifts in parts per million, ppm, shown for illustration), but other characteristics could also be used especially at higher levels (e.g. intensity ratios). Groupings in the metasignature reflect the information obtained by using the COSY crosspeak to relate features in the ^1^H 1D data

### Features

In NMR, there are several levels of information. First, data points representing signal need to be separated from noise or background. This represents the most basic level of information. Likewise, information is added when a feature is defined; i.e. boundaries and characteristics are applied to a part of that signal, which is then recognized as a meaningful spectral element that can serve as an independent unit and as a basis for spectral comparison. For annotation in typical metabolomics studies, a feature must also be recognizable by one or more of its characteristics across more than one sample. Note that, in the case of a single spectrum, this mapping is implicit in spectral matching. Figure 3 illustrates examples of the types of structural relationships encoded by each feature type from different NMR experiments commonly used in metabolomics. Features gain complexity moving from left to right. As a concrete example using the molecule butanone, we give the common characteristic of chemical shift, or position, for features of each type in Table 1.

The simplest feature, a resonance, represents a Fourier-transformed signal from magnetically equivalent nuclei distributed about a frequency with a Lorentzian or Voigt-like shape (Marshall et al., 1997). Multiple resonances can result from chemically equivalent nuclei that exist in magnetically distinct environments because of spin–spin coupling (Hoye et al., 1994) (Fig. 3, pink ellipses). Resonances are typically described by five characteristics: frequency (at maximum; e.g. 2.42 ppm in Table 1), height (at maximum), Full-Width at Half-Max (FWHM), line shape and signal-to-noise ratio, but a frequency range is sometimes also given (see Fig. 4 for computational characteristics).

In 2D NMR, the basic feature is a crosspeak (2D resonance) resulting from the relationship between distinct nuclei (colored connections in Fig. 3). A crosspeak falls on the respective axes at the resonant frequencies of each nucleus being measured, and its existence directly indicates a specific molecular relationship between two nuclei depending on the type of experiment. The information provided by a crosspeak can be the association of two distinct nuclei across multiple bonds (HMBC and COSY), within a spin-coupling system (TOCSY), or between directly bonded nuclei (HSQC; INADEQUATE). A critical point is that the basic feature in 2D data marks a structural relationship between nuclei. Additionally, 2D relationships can be homonuclear or heteronuclear. Depending on the resolution and type of the experiment, the same resonance characteristics discussed above can be measured with the advantage of being resolved into an additional dimension.

### Peaks and compound features

While 2D features report on structural relationships directly, comound features are needed to accomplish the same task in 1D data. To this end, there are two routes to describing more complex elements of a spectrum which correspond to two directions of information flow (Fig. 4). A spectroscopist can recognize a group of resonances as a peak if it is known to be a part of a signature, or using expert judgment. However, this implies a known connection between spectral elements and molecular structure and this information is often computationally inaccessible. Alternatively, clear patterns can be used to build compound features from simpler ones. On one hand, the features derived from higher levels of knowledge are more reliable because they make fewer assumptions about, for example, feature grouping. However, it may still be advantageous to build features of increasing complexity without this knowledge because they can then be described using more detailed characteristics. The terms ‘peak’, ‘resonance’, and ‘feature’ reflect these differences; their conflation causes a great deal of confusion in practice. Here we favor the NMR definition of a peak: a cluster of one or more resonances derived from chemically equivalent nuclei (e.g. colored protons in Fig. 3).

#### Simple peaks

Peak shapes are derived from splitting patterns of varying complexity (Hoye & Zhao, 2002; Hoye et al., 1994). Frequency differences (in Hz) between specific pairs of constituent resonances provide J-coupling constants, which are influenced by several aspects of the electromagnetic relationship between coupled nuclei, such as total bond distance, bond angle, and other effects. Coupling type, a description of the observed splitting pattern of a peak, is commonly reported (Wishart et al., 2022) as is the number of observed resonances comprising the peak (e.g. “triplet”). Idealized simple peaks exhibit predictable, symmetric resonance intensities. Additionally, peaks representing coupled nuclei share coupling constant(s), which can be used to confirm a coupling relationship between peaks in simple cases, and can act as a guide in more complex cases.

#### Complex peaks

Complex splitting can produce unique characteristics which can be utilized in annotation, including symmetry, splitting pattern complexity, and strong coupling effects (e.g. ‘roofing’). Often these peaks are characterized more broadly by center frequency, maximum height, and frequency range, or (most often) by a group of these measurements inherited from constituent resonances (e.g. a collection of frequencies). Lastly, peaks can exhibit specific positional variations (center frequency variation) due to several factors such as pH or metal ion concentration in the local chemical environment (Tredwell et al., 2016). Importantly, a peak which shifts due to these effects will shift as a whole unit with its shape unchanged (e.g., the apparent doublets in Fig. 1).

#### Peaks vs. compound features

From the computational perspective, when analyzing an unknown feature, peak-level information is rarely known and must be hypothesized based on patterns observable in the data itself. When peaks cannot be well-defined (due to intra- or extra-molecular overlap, or complex patterns), heuristics can still be used to derive hypothesized groupings of resonances for higher-order matching and assignment tasks (Cobas et al., 2013; Golotvin et al., 2002; Hoye & Zhao, 2002; Hoye et al., 1994). Here we will refer to the resulting spectral elements as compound features (Fig. 4; Table 1) to reflect their different origins from known peaks.

### Signatures and metasignatures

The signature of a compound is its spectroscopic profile in a single spectrum for a given experiment type. In other words, it is what would be observed if a pure solution of the compound were measured. However, there are several ways to describe and derive a signature computationally, and these differences are important when comparing spectral profiles. For example, a hypothetical signature can be obtained from a STOCSY analysis (Cloarec et al., 2005), but this would carry different information compared to a reference spectrum of a pure compound, since signals from distinct compounds might share high statistical correlations. Furthermore, even with noise excluded, the full signature is not necessarily the ideal database query or descriptor. Complex overlap, and variable chemical shifts (∂) are not well-captured by a signature which is simply represented as a full-resolution vector. Instead, information needs to be extracted in the form of features at different levels to maintain flexibility when necessary, and the collection of these features together can form a more useful, derived signature (such as a set of “peak-picked” resonances, e.g. Table 1). This process involves data reduction, and needs to be done carefully so as to not exclude useful information. We use the term sub-signature to describe any subset of compound or simple features extracted from a signature.

Likewise, the concept of a signature can be extended to the collection of features attributed to a given compound across experiment types on a single or multiple samples. We refer to this as a meta-signature. Meta-signatures are a familiar but abstract concept, as they are implicitly produced when a spectroscopist aligns crosspeaks in 2D spectra to glean interatomic relationships (represented as a multigraph in the top right of Fig. 4). While a signature can be conceptualized as purely signal (in the case of pure compound spectra) or derived features, a meta-signature integrates the features observed across experiment types, and this integration necessitates feature definition and extraction at some level. This usually means relating features from different experiments based on common dimensions of their characteristics (e.g. aligning spectra based on a common ∂^1^H axis). For example, crosspeaks representing ^1^H–^13^C bonds in an HSQC can be linked using multi-bond connections observed in an HMBC (Fig. 4), or a COSY crosspeak can be used to link two coupled peaks (Table 1). Meta-signatures can therefore tie together components of a signature which may otherwise appear unrelated. We note that, although metasignatures are typically derived from 2D data, STOCSY analysis on multiple samples can also contribute feature connections in 1D data (Fig. 4; discussed below).

## Extracting features, compound features, signatures, and subsignatures

Feature characteristics can be derived from different sources and approaches. Resonance characteristics, for example, are commonly extracted using peak-picking algorithms, binning/bucketing algorithms (Sousa et al., 2013), and approaches which attempt to deconvolute to individual resonances or reduce overlap in other ways (Zeng et al., 2020). While the latter would be preferable, deconvolution of NMR data is still an open problem in complex mixture analysis. As a result, extraction of all features should not be expected. Nonetheless, we detail some of the approaches used to extract characteristics for each feature type when possible.

### Bottom-up: data-driven feature extraction

#### Simple features

In complex mixture data from experimental samples, features are usually extracted directly using data reduction approaches, including binning, peak-picking, and deconvolution. These approaches address common challenges. First, the issue of positional variation (the alignment or correspondence problem) is addressed by alignment algorithms (Vu & Laukens, 2013; Vu et al., 2011) and binning to combine a fixed or dynamic number of adjacent data points to capture the same resonance maximum across multiple spectra (Sousa et al., 2013). Binning yields frequency (∂, ppm) and intensity in the form of signal maximum or integrated area under the curve (AUC). If the spectra are well-aligned or grouped, resonances can be “peak-picked” using a variety of approaches such as local maximum above noise threshold (Koradi et al., 1998), or wavelet filters (Beirnaert et al., 2018; Du et al., 2006; Trbovic et al., 2005). These algorithms tend to be packaged with other utilities, or scripted in-house and do not often receive focused attention. Lastly, resonances can be obscured by overlap—a situation where two or more resonances are overlapped such that apparent resonance characteristics are altered or altogether masked (e.g. Fig. 1). This tends to be more of an issue in ^1^H spectra due to the narrow dispersion of proton frequencies for small molecules (~ 0–12 ppm) compared to ^13^C frequencies (~ 0–200 ppm).

Deconvolution algorithms attempt to remedy this issue by decomposing a spectrum into its constituent features. However, the true number of resonances is usually unknown, and varying numbers of resonances can often fit the data equally well, requiring assumptions to be made about resonance characteristics to break this degeneracy (Cobas et al., 2013). A large amount of NMR signal collected in metabolomics experiments often goes unused as a result of inability to define features in overlapped regions. An interesting approach in this area is the complete reduction to amplitude-frequency table (CRAFT) approach, which extracts deconvoluted frequency and position characteristics from time-domain data and obviates several spectral processing steps (Krishnamurthy, 2013).

### Compound features from 1D data

In 1D mixtures a fundamental issue for annotation is knowing if several features belong to the same molecule. In some 1D cases, compound features can be built up from resonances when differences in chemical shift are hypothesized to be J-coupling constants. Likewise, if splitting patterns are simple enough, then the expected signal ratio between peaks or overall peak shape (including symmetry, roofing, etc.) can be used to verify the connection between resonances and allow recognition of a compound feature. Early attempts at compound feature/sub-signature extraction emerged from Computer-Assisted Structure Elucidation (CASE) research, which focused on assignment of spectral features in combinatorial synthesis reactions with known structural motifs (Rossé et al., 2002). PROOFSTR recursively estimated splitting trees from 1D ^1^H spectra while accounting for multiplet shape and symmetry (Golotvin et al., 2002). Likewise, MestreNova produced a J-coupling constant extraction tool (Cobas et al., 2005), and later developed an automatic assignment that extracts peak position, number of nuclei, and multiplet shape as a dimensionless “pure-shape characteristic” from the reference and experimental spectra (Cobas et al., 2013). However, these compound features must be considered cautiously, as the assignment of a compound feature to a physical mechanism (e.g. scalar coupling) cannot always be inferred with confidence from the mixture data alone.

### Compound features from 2D data

For 2D multibond correlation experiments (COSY, HMBC), compound features can be built from basic spectral elements more reliably (Figs. 2 and 3, moving from left to right); that is, 2D peaks can be directly agglomerated into compound features by connecting pairs of crosspeaks which share a frequency on either axis. In heteronuclear spectra such as ^1^H-^13^C HSQC, crosspeaks typically do not line up, as chemically distinct protons are less often bonded to the same carbon. Even though compound features obtained from 2D data are generally high-confidence because the data communicate bond information directly, orthogonal data are still typically needed to connect them in mixture data. Spin systems are often broken up in a molecule by a ‘silent center’, an atom lacking direct bonds to protons (Dona et al., 2016). In both 1D and 2D data, these silent centers prevent connecting nuclei across the entire molecule, meaning that their corresponding subsignatures are disjoint (e.g. the pink TOCSY signature in Fig. 3) and must be joined by data from other experiments. Alternatively, compound features can be derived from empirically or statistically sourced full signatures (sometimes obtained by a meta-signature or a collection of 1D spectra; see below).

### Signatures and metasignatures are connecting points

Signatures themselves can be derived from four main sources. First, a spectrum of a pure compound can be acquired under controlled conditions, as found in reference databases. While such a signature is less complex than mixture data, features must still be extracted from it to do feature-based matching. These features (assuming no impurities or artefacts) are known to be in the same molecule, but, particularly in 1D cases, the difference between compound features and peaks typically remains unless such annotation is provided from another source (i.e. expert knowledge or other data).

Second, a predicted or computed spectrum can provide a range of signature information. In fact the Hierarchical Organization of Spherical Environments (HOSE) methods (Bremser, 1978) and others provide quite accurate ^13^C 1D spectrum predictions, and several approaches for ^1^H 1D prediction have also been developed (Cobas et al., 2013; Dashti et al., 2017, 2018; Smurnyy et al., 2008). Prediction from structure is powerful in theory, because it does not rely as heavily on acquisition parameters, it is more scalable than collecting high-quality experimental data, and provides a first-principles, structural basis to link features to signatures or metasignatures. This offers a great deal of flexibility for matching. Recently, NMR spectrum-based chemical similarity networks which delineate substructure-subsignature releationships have been published (Egan et al., 2021; Flores-Bocanegra et al., 2022; Reher et al., 2020), and offer several opportunities. As these become more widely used, modeling steps could be re-used to expedite modeling of new compounds. Additionally, model updates and useful metadata could be propagated intelligently to compounds in the network, or to compounds in similar natural product compound networks (Kim et al., 2021).

Third, a metasignature can easily be decomposed into signatures. There are two key advantages to sourcing signatures from metasignatures: the signature is supported by orthogonal data types, and high-quality peak information may also be obtained. Previously disjoint compound features (e.g. blue HSQC connections in Figs. 2 and 3) can be linked together by a metasignature (combined connections in Figs. 2 and 3). Then, this information can be carried back down to the signature (i.e. single experiment) level as a high-quality signature (Fig. 4). This latter step is critical because most reference databases do not contain meta-signatures across multiple experiment types; rather, they contain signatures for a given experiment type derived either from pure compound spectra or integration into a metasignature (e.g. Bingol et al., 2012, 2014; Robinette et al., 2008). Meta-signatures themselves can be derived from 1 and 2D data, but generally not from 1D spectra alone. Potential exceptions, albeit much less common in metabolomics settings, include DEPT and ^1^H-detected 1D HSQC/HMBC experiments, as bond information between nuclei is conferred in a 1D data type. For 2D multi-bond correlation experiments, such as long COSY (Dona et al., 2016; van der Hooft & Rankin, 2016) or HMBC (Bakiri et al., 2018), a complete signature can be built directly from the data when compound features share a chemical shift and all features are linked.

Lastly, in the case of complex mixtures, correlation-based dereplication approaches can extract compound signatures from a collection of NMR mixture data from different samples. The main statistical dereplication methods relevant to annotation include STOCSY (Cloarec et al., 2005), CLASSY (Robinette et al., 2009), SHOCSY (Zou et al., 2014), and STORM (Posma et al., 2012). For example, the Metabomatching suite uses three statistical methods for obtaining signatures, and then compares them (Khalili et al., 2019). Statistically derived signatures should be used with caution, as they commonly include artifacts or missing peaks, do not provide relative quantification, and are sensitive to overlap.

### Top-down: high-quality subsignatures from signatures

Compound features must be taken as hypotheses when derived directly from complex mixture features and without incorporation of higher-level information. However, when a signature is available (either experimental, statistical, or computed from first principles) it can be broken down into related compound and simple features, or subsignatures typically based on spin systems. In simple 1D cases, modeling is often used to generate a signature and subsignatures. For example, the ChenomX suite includes a pH-dependent reference library which integrates subsignatures based on experimental data and modeled spin-coupling relationships (Mercier et al., 2011). Likewise, the GISSMO library provides a spin system matrix containing all pairwise coupling constants for each compound. Peaks and their profiles are then simulated for any field strength; furthermore, spin systems are compactly represented as frequency differences between resonances (Dashti et al., 2017, 2018). Alternatively, any method yielding compound features could also be applied to a signature.

2D signatures allow for more confident subsignature extraction. Demix demonstrated PCA-based demixing of 1D traces of TOCSY spectra with varied mixing times, where principal components yielded subsignatures corresponding to spin systems (Zhang & Brüschweiler 2004). This method connects data points into subsignatures on the basis of their co-occurrence in a meta-signature derived from statistical integration of multiple spectra collected on the same sample. On the other hand, DemixC decomposes single covariance-processed 2D ^1^H-^1^H TOCSY spectra into spin systems directly using a clustering approach, which allows it to be more robust against overlap but provides less-confident compound feature definition (Zhang & Brüschweiler, 2007). 2D signatures can also be projected to 1D for broader utility. Bingol et al. (2012) did this for ^13^C–^13^C TOSCY, 2D ^13^C–^1^H HSQC-TOCSY, and 2D ^1^H-^1^H TOCSY data (Bingol et al., 2014), and subsignatures were then compiled into the TOCCATA (TOCSY Customized Carbon Trace Archive) database and ^1^H(^13^C)-TOCCATA databases, respectively. Maximal clique-based subgraph extraction was also used to build spin systems from 2D ^1^H-^1^H TOCSY data, yielding complete spin–spin connectivity information (Li et al., 2017). Compound features, like those obtained using ^1^H iterative full-spin analysis (HiFSA) simulations, require precise reporting (Pauli et al., 2014), but allow for more thorough feature characterization (e.g. Pauli et al., 2016) and even modularized structural subsignatures (Napolitano et al., 2015). Lastly, MADByTE utilizes metasignature information derived from TOCSY and HSQC data to pull out “spin-system features”, which are then compared (by chemical shift) with experimental data (Egan et al., 2021; Flores-Bocanegra et al., 2022).

## Matching: leveraging computational characteristics

The information underlying different types of features determines the computational approaches that can be used for comparison with reference databases in the annotation process. We next give examples of the use of different types of information in database matching, and note important algorithms used. Our discussion is intended to be illustrative rather than comprehensive. Once information has been extracted from the data, spectral comparison to reference data can take place. It must be noted that currently available reference databases are extremely limited in their coverage of the space of all possible metabolites, and the representation of molecules tends to be biased towards those found in the most common sample types (e.g. blood serum, urine, or tissue extracts). Database search styles for spectral data can be divided into three main types, each distinguished by its output (Mohamed et al., 2015; Zürcher et al., 1988). Either (1) an exact match can be sought (with no flexibility), or (2) results most similar to or consistent with the query data can be returned in a ‘ranked’ approach; this is the search method most groups have pursued. Alternatively, (3) an ‘interpretative’ search can provide a set of subsignature-specific matches, which allows a compound structure to be deduced from the combination of subsignatures. (Mohamed et al., 2015) argue that, although an interpretative search is feasible for ^13^C data (Koichi et al., 2014), it is not tenable for 1D ^1^H NMR because of overlap and other issues. However, several recent tools have challenged this notion by factoring in subsignatures, or subsignature-substructure relationships, during matching (Charris-Molina et al., 2020; Wang et al., 2019). Such approaches may hold promise in mitigating the issue of reference database coverage and bias by incorporating more information while allowing partial matches to subsignatures that may be shared between metabolites.

Similarly, matching algorithms leverage the advantages of different data types. There are different types of spectral comparisons that can be made to database entries (Mohamed et al., 2015). We point these out, and build on this framework to include different forms of information which can be extracted from common NMR experiments. Indeed, much of the variation between reference-based annotation programs results from the choice of information used for matching.

### Comparisons between feature lists

Common search functions (Cui et al., 2008; Robinette et al., 2008) formulate matching as an assignment problem, and employ the Hungarian algorithm as the core computational workhorse. This classic bipartite weighted graph assignment algorithm permutes the cost (or peak distance) matrix calculated between lists of resonance frequencies to minimize its trace (Kuhn, 1955). One downside to this approach is that, when the query and reference lists have very different sizes, the overall score is not penalized by unmatched peaks. To address this, Robinette et al. applied the algorithm iteratively, eliminating previously matched peaks in each iteration to force (dummy-matched) peaks to be matched with worse scores. The sum total distance of a match is then used for ranking matches (Bingol et al., 2012, 2014; Robinette et al., 2008). This ranking strategy is well-suited for empirically-derived queries such as those obtained from 2D data using e.g. DemixC (Robinette et al., 2008) or TOCCATA (Bingol et al., 2012, 2014), or when reference peaks exceed the number of observed query peaks. However, it is not optimal for the peak lists obtained from statistical deconvolution/dereplication approaches, which are typically rich in false-positives and require more flexibility. In other words, though the match computation is the same, the meaning of a match is different when a query can be assumed to be devoid of false positive signals. Finally, once peaks are matched, the Tanimoto (Jaccard) coefficient can be used as a scoring function that accounts for the number of expected peaks vs. matched peaks (Mohamed et al., 2015). Although not extended yet to complex mixtures, DP4-AI uses an innovative probabilistic matching mechanism that is an extension of the Hungarian algorithm and gives an overall fit metric (Howarth et al., 2020).

### Compound feature/subsignature-driven comparisons

Importantly, while the sum of an independent set of subsignatures and/or resonances looks the same as a signature, there is a difference between the two when it comes to matching. The question of how well a small set of linked subsignatures fits a small set of reference features is much more constrained than the question of which features, if any, correspond to that small set of reference features. This constraint has important implications for false positives as well as computational tractability. Positional variations require flexibility, which necessarily introduces degeneracy and increases the effective number of sub-hypotheses that need to be tested to assess a match. This increases the chance of false positives at the feature matching stage, which will translate to false positive annotations with opaque justification. Subsignatures, therefore, are perhaps the best basis for flexible matching. Compound features can also be used; they are less reliable, but may afford similar flexibility for compounds which only have 1D data in reference databases and do not benefit from high-quality peak information derived from expert annotation or 2D data.

Likewise, while resonance-level information is more granular, it often lacks information needed for dependable matching. Various aspects of 1D ^1^H data have been used for matching, including peak position, but also line shape, symmetry, and J-coupling constants. These characteristics were incorporated into a single bespoke match factor for similarity to spectra predicted from structural motifs (Golotvin et al., 2006). Likewise, MestreNova developed an automatic assignment approach that checks annotations by comparing predicted and experimental multiplet pairs on the basis of peak position, number of nuclei, and the multiplet “pure-shape characteristic” (Cobas et al., 2013).

ChenomX fits peak subsignatures to experimental data and allows them to move independently in an interactive manual fitting process. The software also provides an automated fitting function driven by a genetic/simulated annealing algorithm that incorporates the shapes, positions, and resonance widths of each reference multiplet peak cluster for a given 1D ^1^H spectrum (Mercier et al., 2011). However, it is important to note that fits are computed for individual experimental 1D spectra. Therefore, matching only relies on the data for one spectrum and does not utilize information from other sources, such as correlation dereplication methods (Note: the algorithms underlying the software have likely developed beyond this point, but we are unaware of literature detailing those updates).

The COLMARm query system uses subsignatures and their 1D projections for computational demixing and annotation (Bingol et al., 2012, 2014; Robinette et al., 2008). Users upload up to three types of 2D spectra for the same complex mixture sample, and receive automated annotation of those spectra based on several databases with minimal input. The large number of high-quality reference spectra, the accuracy of the annotations, and the interface provided make this a very popular tool set.

Other methods employ spin-system extraction approaches driven by 2D data (Li et al., 2017; Napolitano et al., 2015) allow comparison (by lists of chemical shifts) to database subsignatures, and even enable substructure spectra as building blocks for larger molecules (Napolitano et al., 2015).

### Full signature comparisons

Full spectral signature comparison methods allow the most flexibility by capturing the “broader picture” of a signature. These can be integrated with information from lower levels during or after fitting (as filtering steps), and are broadly organized into those which handle reduced signatures (to linked subsignatures or features), and those which utilize full-resolution data directly. A signature as a set of linked features, can be compared using any of the characteristics of those features. However, the integration of this information can be difficult to interpret/control, and can be implicit in the scoring mechanism used, like the Tanimoto (Jaccard) measures (Mohamed et al., 2015).

For full-resolution treatment of signatures, (Mohamed et al., 2015) lay out three classes of spectral comparisons for numerical vectors: correlation-based, distance-based, and tree-based metrics. The latter is less commonly used, and is rooted in the idea of a balance of signal mass across the spectrum, to allow flexible global pattern matching. This approach leverages the intuition that similar molecules have generally similar spectral properties (Castillo et al., 2013; Zürcher et al., 1988), and would be useful for comparing signatures which have positional differences at the local level. However, criteria such as matching coupling constants may need to be screened downstream. Additionally, model-based metabolite fitting and quantification methods, such as BATMAN (Hao et al., 2014), BAYESIL (Ravanbakhsh et al., 2015), and ChenomX (Mercier et al., 2011) are inherently full signature comparisons, and they differ from the metrics discussed above.

Like the fitting methods, deep learning and pattern recognition-based (Dubey et al., 2015; Hubert et al., 2014; Napolitano et al., 2013; Wolfram et al., 2006) methods, in theory, use optimal weighted combinations of spectral information across all levels to satisfy the training goal, whether that be sample classification, molecular classification, identification of substructures, or distinguishing molecules and even isomers. SMART (2D, Zhang et al., 2017) and SMART 2.0 (1D, Reher et al., 2020) approaches use deep learning to learn substructures which distinguish compounds; however, the degree of integration of these as a whole signature is unclear. Finally, another deep learning tool which classifies natural products based on molecular substructures may assist in exploring this direction further (Kim et al., 2021). The major challenge here is the availability of high-quality labelled training data; however, this has been remedied by the Siamese network architecture, which performs well for data with few training examples per class (Zhang et al., 2017). Depending on the nature of the training data (complex or simple mixture data), deep learning methods could conceivably even account for extramolecular spectral cues such as matrix-related effects and biases. It is important to consider these potential influences as more deep learning and full-signature methods are employed.

Much of the recent work in signature-based comparison comes from advances in forward and reverse prediction of ^13^C reference spectra, particularly when the molecular formula is known. Imitation learning was used to generate molecular graph topologies consistent with chemical shifts and peak splitting in a ^13^C 1D NMR signature of an unknown (Jonas, 2019). Another, iterative algorithm builds a tree of structures consistent with the experimental signature using a set of constraints and a de-novo molecule generator. Reference spectra for the molecules are computed using quantum chemistry, and are compared to the signature using the Wasserstein distance (Zhang et al., 2020). In another approach, ^13^C NMR queries are matched directly to HOSE- and MPNN-predicted chemical shifts of candidate molecules using cosine similarity (Kwon et al., 2021). Finally, an ML-driven model was recently trained to recognize hundreds of substructures in 1D ^13^C NMR data, which can then be used for automated structure elucidation. The model uses different pooling layers with the idea of optimizing feature and compound feature characterization separately. The method can also work with ^1^H data (Huang, 2021).

We are not aware of any platform that utilizes meta-signatures explicitly; this may be accomplished using multigraph methods or weighted combinations of independent match scores for each and may be a fruitful avenue of future research. However, (Joesten & Kennedy, 2019) outline a general formalized approach to using metasignature information for ranking putative annotations manually. Likewise, (Jonas, 2019) uses a Markov decision process approach to build molecular graph topologies from ^13^C chemical shifts and splittings. A few subsignature-based methods do simultaneously match resonances in multiple spectrum types, including COLMARm (Bingol et al., 2014), and MADByTE (Egan et al., 2021; Flores-Bocanegra et al., 2022), and graph-based comparisons are used in connectome research (Frigo et al., 2021).

## Assessing and communicating confidence

### Ranking hypotheses

Algorithms for matching an experimental signature to a reference database will almost always return matching results for a given query, as the matching process generally produces at least one (true or false positive) match when sufficiently large reference databases are used. However, for these results to be practically useful, users need an estimate of the matching confidence to filter and rank each potential annotation. This way a decision can be made on further use (e.g. in pathway analysis) or to go forward with confirmatory experiments. A simple proxy for confidence is the match score, which quantifies the goodness of fit between the query signature (e.g. list of chemical shifts) and the matching reference. So how can this be done for matches generated from features of different complexity, or feature lists of different sizes?

The simplest metrics for goodness of fit involve the proportion of observed to all possible features matched within a tolerance (typically ∂ at feature max); this is given by the Jaccard (or Tanimoto) index which has also been applied to chemical structure fingerprints (Bajusz et al., 2015). Total distance between the best-fitting features in lists can be used, where unevenly sized lists are penalized (Robinette et al., 2008). Intensity differences can also be included, but this introduces the issue of ensuring comparable intensities between the query and reference. Generally, a good starting concentration is the maximum allowed such that no reference signal exceeds a matching query signal (Hao et al., 2014).

Metrics useful for signatures and compound features allow for more detailed comparisons using higher-order information, such as peak shape and accounted-for signal. Spectral similarity metrics differ across studies. Strength of similarity can be used to rank annnotations; although in general, p-values from statistical tests associated with correlation-based metrics should not be taken as accurate as spectral data are not independent. It may be more useful to express confidence as signal accounted-for, e.g. using root-mean-square error (RMSE), or Wasserstein distance (Zhang et al., 2020). Composite scores can also incorporate other scores (e.g. proportion of expected peaks matched), each of which must be given an appropriate weight which is hard to determine. Furthermore, if compound features or subsignatures are matched independently, a partial signature match may still be worth reporting. Irrespective of the score used, it can be hard to decide between many highly ranked hits, and setting a threshold on the score for an acceptable match is extremely problematic.

### Controlling false positives

One approach is to find a threshold which limits the rate of false positive annotations, by estimating the distribution of the match score under a suitable null hypothesis. The decoy database is a key idea in this direction, which has been widely used to estimate annotation confidence in mass spectrometry-based analysis (both proteomics and metabolomics) but has not yet received much attention in the NMR annotation field. In this approach, one generates a reference database composed of artificial entities (peptides, metabolites etc.) which are as similar as possible to the true reference database, while being clearly identifiable as incorrect hits. For example, in proteomics, one can use reversed sequences to produce a decoy database of peptides with similar size and amino acid distribution to the reference, yet where each entity cannot be a correct annotation. A simple approach in NMR could involve constructing decoys by picking peaks at random from the reference database, in such a way as to maintain the reference distribution of numbers of peaks per compound. This approach has been applied in MS metabolomics (Elias & Gygi, 2010; Scheubert et al., 2017) and improved upon by using fragmentation tree information and peak co-occurrence. This latter idea also seems straightforward to apply to other spectroscopic data, but it remains to be seen whether decoy databases can provide reliable confidence estimates for NMR annotation. 

Whether one uses the decoy idea or not, it is clear that the nature of the reference database will heavily influence the confidence of any annotation. In any database matching problem, a larger reference database increases the chances of a false positive match. This is a well-known problem in sequence based bioinformatics (e.g. BLAST search) where p-values are converted to E-values to account for this effect. On the other hand, the chance of a false negative (i.e. lack of any match) is increased with smaller or incomplete databases, which will nearly always be the case given the vast chemical diversity of potential metabolites. Thus, for realistic matching strategies, there will always be a balance between recall (ability to make any match) and precision (accuracy of the match), and larger databases will require higher fidelity matching to avoid rampant false positives. Beyond just size, the composition of the database will also have a large impact. For example, a database focused on compounds known to be present in normal human blood may be excellent for annotation of plasma derived spectra, but contribute many false positives and negatives when used to annotate samples of a different type.

One aspect of database heterogeneity that can be useful in scoring or confidence estimation is the notion of peak uniqueness. A peak is said to be unique for a given database if there is no other compound with a peak at the same chemical shift. Any match to a unique peak could be considered strong evidence that the reference compound is present (assuming the other reference peaks are also observed). This uniqueness could be taken into account in match scoring or confidence estimation, perhaps by upweighting highly unique matches. The idea can be extended to unique patterns of multiple peaks which has a clear application when scoring reference compounds with identical signatures (Tulpan et al., 2011). It also has the advantage that the weighting can be calculated automatically for any database and does not depend on human interpretation. To summarize, it is currently the case that there is no standard way to estimate confidence of a match. However, there are several ideas which could be applied and which are independent of the matching algorithm itself.

## Overall conclusions

Computational annotation accelerates the identification process by suggesting high-quality hypotheses that can then be tested computationally and experimentally. These processes fundamentally rely on the various layers of interconnected information contained within and across NMR spectra. Therefore, it is necessary to have a clear understanding of how data are interpreted for structural information, as well as how they are handled in computational comparisons. By viewing the wealth of annotation software available to the NMR community through these lenses, several key points emerge:

- Reduction of spectra to individual sets of features may not be as straightforward as it first appears. The same data points can be interpreted in a range of ways.
- The optimal comparison to a reference signature is likely to take place at similar information levels for both experimental and reference signatures/subsignatures. Matching at higher levels allows for flexibility and is expected to be more reliable as meaningful features (e.g. peak shapes) are being compared.
- Matching algorithms must be appropriate for each data type, and scoring metrics can exert subtle influences on rankings and performance quality.
- Significant progress has been made recently in the incorporation of powerful statistical methods like deep learning in the interpretation and prediction of NMR spectra, from features to signatures.

As the field progresses, reporting of annotations, and confidence in them, should be based on the structural information employed in the annotation process. Each level of information provides a different form of evidence for an annotation, and it is expected that this multifaceted approach to the problem will be increasingly used as the field moves forward.

## Data Availability

Data sharing not applicable to this article as no datasets were generated or analysed during the current study.
